# The Most Recent Insights into the Roots of Gastric Cancer

**DOI:** 10.3390/life14010095

**Published:** 2024-01-08

**Authors:** Lorena Elena Meliț, Cristina Oana Mărginean, Reka Borka Balas

**Affiliations:** Department of Pediatrics I, “George Emil Palade” University of Medicine, Pharmacy, Sciences and Technology, Târgu Mureș, Gheorghe Marinescu Street, No. 38, 540136 Târgu Mureș, Romania; lorena.melit@umfst.ro (L.E.M.);

**Keywords:** *Helicobacter pylori*, gastritis, gastric cancer

## Abstract

*Helicobacter pylori* (*H. pylori*) is the most common bacterial infection worldwide, usually being acquired during childhood, and its persistence into adulthood represents one of the main contributors of gastric carcinogenesis. Based on these statements, it would be of great importance to know if the most early premalignant transformation occurs in children or later since, this would enable the development of effective anti-tumorigenesis strategies. The interplay between *H. pylori* virulence factors, the host’s responses modified by this infection, and the gastric microecology are complex and eventually lead to the development of gastric cancer in susceptible individuals. Several biomarkers were identified as major contributors of this long-lasting process, such as pepsinogens, gastrin 17, lipid-, glucose- and iron-metabolism parameters, immunity players, aberrant bacterial DNA methylation, *H. pylori* virulence factors, and hallmarks of gastric dysbiosis. Several of these biomarkers were also identified in children with *H. pylori* infection, independently of the presence of premalignant lesions, which were also proven to be present in a subgroup of *H. pylori*-infected children, especially those carrying extremely virulent strains. Therefore, the most incipient premalignant gastric changes might indeed occur early during childhood, opening a promising research gate for further studies to delineate the border between infection and cancer.

## 1. Introduction

*Helicobacter pylori* (*H. pylori*) is the most common microbial agent involved in the development of gastrointestinal infections in both children and adults; it is usually acquired during childhood and, with a lack of treatment, it might persist for life. Its persistence triggers important pathological changes within the gastric mucosa, beginning with chronic gastritis and ending with gastric cancer, with intermediary stages like atrophic gastritis, intestinal metaplasia, and dysplasia [1]. In fact, the intermediary stages between the initial unspecific gastric inflammation and gastric cancer might be the best opportunity to stop the carcinogenesis’ process. Although gastric cancer is a rare entity in children, *H. pylori* positive chronic gastritis is one of the most common reasons for presenting to the pediatrics gastroenterologist [2]. Moreover, the gastric mucosal damage induced by *H. pylori* progresses from childhood into adulthood, eliciting several inflammatory responses, which involve the participation of polymorphonuclears, macrophages, and B- and T-lymphocytes, along with the synthesis of several proinflammatory cytokines [3,4]. These inflammatory responses are responsible for the chronic transformation of gastric inflammation with a slow progression towards gastric cancer due to the release of nitrogen species and reactive oxygen from the activated inflammatory cells, and the subsequent promotion of cellular apoptosis and DNA injuries [5]. Eventually, the long-term persistence of *H. pylori* and the chronic inflammation of the gastric mucosa triggered by this bacterium will lead to the activation of a wide spectrum of oncogenic pathways [6].

Although *H. pylori* infection represents one of the main causes of gastric cancer, the process of carcinogenesis is much more complex and involves the contribution of several factors related to both the bacterium and host, as well as the environment [7].

As previously mentioned, the cytokine secretion induced by the persistence of *H. pylori* is one of the main steps in the progression of chronic gastritis towards gastric cancer. Thus, several cytokines were proved to be related to the occurrence of gastric carcinogenesis such as interleukin (IL) 1, 6, 8, 10, 18, tumor necrosis factor alpha, and transforming growth factor beta [4]. The precise role of certain of the previously mentioned cytokines in promoting or suppressing carcinogenesis seems to rely on the host’s peculiarities, such as gastric microenvironment. A recent review of our team indicated that the wide diversity and abundance of gastric microbiota represent protective factors against *H. pylori* infection [8]. In addition, the same review pointed out that, in pediatric patients, certain gastric resident bacteria such as *Neisseria*, *Streptococcus*, *Staphylococcus* and *Rothia* have the ability to establish a partnership with *H. pylori* in order to augment the subsequent inflammatory responses [8]. Since the majority of the studies focused on assessing gastric microbial composition in subjects with *H. pylori* infection, few data are available regarding the healthy uncolonized adults, which revealed that they harbor a predominance of six bacterial genera: Streptococcus, *Prevotella*, *Oribacterium*, *Megaspherae*, *Propionibacterium* and *Capnocutaphaga* [9,10,11,12]. 

Postbiotics are defined as compounds of inanimate microorganisms, either combined or not with their components, which were proven to have a benefit for the host’s wellbeing, with recent evidence also suggesting their role in cancer patients [13]. Thus, exopolysaccharides, biopolymers that are released from a different type of bacteria, were suggested to have anti-proliferative effects on different cancer cells or induced apoptosis, making them useful in patients with colorectal cancer or hepatocellular carcinoma [13,14]. Moreover, bacterial cell fragments such as *H. pylori* lipopolysaccharide were also indicated to be involved in carcinogenesis, but their promoting or suppressing effects depend on the type of cancer, the applied substance, the cell line stage and the invasiveness [13,15]. The role of other bacterial components, such as tryptophan metabolites, extracellular vesicles and short-chain fatty acids, were also suggested to play an active role in carcinogenesis due to their anti-tumor properties [13], but their precise role remains to be established in future studies. The role of microbiota and their modulation in promoting or suppressing carcinogenesis was also sustained by the review of Sepich-Poore et al. [16]. 

Previous studies found a close interrelationship between ILs and macrophages, with *H. pylori* as the core of this interplay. Thus, *H. pylori* has the ability to attract macrophages and to activate them in order to synthesize ILs, tumor necrosis factor alpha, GRO-alpha, and MIP-1 alpha, which will subsequently increase the recruitment and activation of supplementary macrophages and T cells [17,18]. Aside from macrophages, *H. pylori* also expresses a chemotactic effect on polymorphonuclear cells due to the neutrophil-activating protein, an important virulence factor [19]. Thus, both macrophages and neutrophils become partners of *H. pylori*, enhancing its persistence within the gastric mucosa and leading to chronic inflammation [19]. Toll-like receptors, acting as the main players in innate immune responses, were also indicated to have an important role in the complex inflammatory array surrounding *H. pylori* [20]. All these findings suggest the presence of a subclinical inflammatory status in *H. pylori*-infected pediatric subject, which seems to be related to the very early onset of gastric cancer. 

The detection and effective eradication of *H. pylori* in the pediatric population represent a cornerstone of gastric prevention. Nevertheless, it is rather challenging for the clinician to choose the most accurate method of detecting the presence of *H. pylori* within the child’s gastric mucosa, mostly because this age group commonly presents with no symptoms, or they present only extraintestinal manifestations. In terms of effective eradication, the most recent reports indicated major progress, since *H. pylori* incidence was recently proved to have a decreasing trend in most geographical areas [21]. Still, *H. pylori*-antimicrobial resistance remains a major concern in pediatric patients, undoubtedly promoting the long-term persistence of this infection, subsequent inflammation, and eventual development gastric cancer. Moreover, it was highlighted that effective eradication might decrease the risk of gastric cancer by 50% [22].

This review aims to assess if the ‘growth of gastric cancer roots’ might occur during childhood.

## 2. *H. pylori* and Gastric Cancer

Gastric cancer is the fifth most frequent cancer worldwide, representing the third leading cause of death due to malignancy [23]. It is well known that, according to the reports of the World Health Organization, from as early as 1994, *H. pylori* was defined as a class I carcinogen, since it seems that approximately 90% of newly diagnosed non-cardia gastric cancers are associated with this infection [24]. Moreover, it was proved that infected individuals have a six-fold higher risk of developing gastric cancer with a lack of effective eradication as compared to those in whom eradication failed [25]. Still, several other factors were emphasized to contribute to the development of gastric cancer, such as family history, lifestyle (dietary habits, smoking, or alcohol consumption), and Epstein-Barr virus infection [26], explaining the fact that not all infected individuals will eventually develop gastric cancer. 

### 2.1. The Interplay between Host- and H. pylori-Related Biomarkers

Although the most accurate diagnostic tool for gastric cancer is upper digestive endoscopy, recent research has focused on assessing the role that several biomarkers play in the early detection of premalignant and malignant transformation and the selection of patients, which would benefit from further investigations. 

Pepsinogens are produced in the host’s stomach and are classified into pepsinogen I, produced by the mucous neck and chief cells within the gastric corpus, and pepsinogen II, synthetized by the Brunner gland and cardiac pylori cells. Important changes in their levels were noticed in patients with perturbations of gastric homeostasis, which is excessively produced during inflammation, and severely decreased in the setting of atrophic gastritis [27]. Several studies emphasized their predictive power for detecting patients with an increased risk of gastric cancer, with the ratio between pepsinogen I and II serving as the most reliable predictor [28,29,30,31]. Interestingly, *H. pylori* infection was found to be significantly associated with increased levels of pepsinogens [32], while individuals with gastric cancer present significantly decreased levels of pepsinogen I and pepsinogen I/pepsinogen II ratio [33]. Moreover, a recent meta-analysis pointed out that pepsinogen I < 30 μg/L, pepsinogen II > 30 ng/mL, and pepsinogen I/II ratio < 3 are significantly associated with gastric cancer [34] (Table 1).

*Gastrin 17* is produced in the antral part of the stomach and has recently been proposed as a predictor for both gastric atrophy and gastric cancer [27]. Thus, an increased level of gastrin was associated with gastric cancer risk [31], although it cannot predict gastric cancer stage [29]. When combined with pepsinogens and *H. pylori* antibodies in the GastroPanel test, the predictive value of gastrin 17 increases [30] (Table 1).

In terms of blood glucose, the evidence is contradictory. A recent meta-analysis revealed no association between this parameter and gastric cancer risk [34]. Nevertheless, Lindkvist pointed out that increased blood glucose levels were related to a higher risk of gastric carcinoma, although only in women [35]. Similar findings were reported by Tran et al., who indicated a higher incidence of gastric cancer in non-smokers, and normal-weight individuals in the absence of a first-class family history of gastric neoplasia [36]. Insulin metabolism parameters were also investigated regarding their relationship to gastric cancer. Thus, Hidaka et al. pointed to a positive correlation between plasma insulin levels, C peptide (only in men), and HOMA-IR and gastric cancer [37] (Table 1).

Lipid metabolism parameters follow the same trends as glucose in terms of controversies. Thus, while Asano et al. concluded that low serum cholesterol might be considered an independent risk factor for gastric carcinoma [38], a meta-analysis of five studies failed to identify any correlation between this parameter and the risk of gastric neoplasia [34] (Table 1).

Based on the risk of occult bleeding in patients with gastric cancer, several authors assessed if iron-metabolism parameters could be used as potential predictors for gastric cancer, suggesting that low iron stores defined by ferritin could represent a reliable early predictor for gastric cancer [34]. In addition, serum iron levels, transferrin, and total iron-binding capacity were also proven to have a certain degree of association with gastric cancer risk [34,39,40] (Table 1).

Gastric microecological dysbiosis is thoroughly discussed in the current research area, and, as previously mentioned, it is definitely implicated in *H. pylori*-associated gastropathies, including gastric cancer development. Thus, certain modifications to the host’s resident microbial community might favor the persistence of this infection, enabling the growth and proliferation of neoplastic cells [8]. Nevertheless, the contribution of gastric microecology to the promotion or suppression of *H. pylori*-derived inflammation probably remains the least-known mechanism in the current era of research. Several recent studies pointed out that other resident bacteria might also contribute, along with *H. pylori,* to gastric carcinogenesis through their ability to reduce nitrates, resulting in the accumulation of N-nitroso and nitrite compounds within the gastric microenvironment, such as *Staphylococcus epidermidis* and *haemolyticus*, *Micrococcus luteus*, *Neisseria mucosa*, *Actinomyces naeslundii* or *Rothia dentocarios*, which dominate the gastric microbial community in children with *H. pylori*-positive gastritis [41,42]. These findings might sustain the hypothesis that the onset of early gastric carcinogenesis is possible during childhood. Although *H. pylori* was defined by some authors as the only ‘true resident’ of gastric microecosystem [9], other commensal bacteria residing in this microenvironment contribute to the acceleration of the inflammation produced by *H. pylori*, subsequently increasing the risk of gastric carcinogenesis [43]. The partnership between *H. pylori* and gastric microenvironment is strengthened by virulence factors of *H. pylori* such as CagA, which was found to interact with *Lactobacillus* to induce additional inflammatory responses, suggesting that lactic-acid-producing bacteria harbored by the gastric microecosystem might be active players, together with the main player, *H. pylori,* in the complex process of gastric carcinogenesis [44] (Table 1).

*MicroRNAs* (miRNAs) were recently emphasized to be potential biomarkers for predicting gastric cancer [45]. Thus, the aberrant expression of miRNAs was associated with gastric carcinogenesis [46,47]. A recent study indicated that miRNA-425-5p, miRNA-1180-3p, miRNA-7641 and miRNA-122-5p could be used to identify the onset of gastric cancer [45]. Moreover, miRNA-135b-5p was found to be significantly upregulated in tumoral gastric tissues when compared to non-tumor samples [48]. The interrelationship between *H. pylori* and the aberrant expression of microRNA expression was sustained by in vitro experiments which revealed that *H. pylori* virulence factor CagA triggers the overexpression of miRNA-543, resulting in the subsequent promotion of cell proliferation, migration to the inflammatory situs, and invasion [49]. According to Liu et al., miRNA-29a/MMP9 follows the same pattern, being overexpressed in patients with *H. pylori* infection as compared to uninfected controls [50]. The upregulation of miRNA-183 was also detected in *H. pylori*-positive gastric cancer patients [51]. Further studies proved that *H. pylori* has the ability to induce changes in more than 50 miRNAs, with a major increase being noticed for miRNA-143-3p [52]. The serum level of certain miRNAs could be used to predict the prognosis of *H. pylori-*induced gastric cancer like miRNA-18a-3p and miRNA-4286, whose overexpression was correlated with invasion, tumor stage and size, as well as lymph node metastasis, which are considered promoters of tumor cell proliferation and motility [53]. Contrariwise, *H. pylori* expressed a downregulating effect on miRNA let-7 via the same CagA virulence factor, which was negatively associated with disease activity and severity scores in patients with gastritis [54,55]. Another biomarker of gastritis activity is miRNA-223, which is related to neutrophil infiltration of the gastric mucosa in the presence of *H. pylori* [54,56]. Additionally, Isomoto et al. highlighted that miRNA let-7b is negatively correlated with IL-1b levels [57]. Animal model studies indicated that miRNA-155-deficient mice developed milder forms of *H. pylori* gastropathies, including gastric atrophy, epithelial hyperplasia and intestinal metaplasia [58]. A similar study revealed that the absence of miRNA-155 expression is associated with impaired antitumor immunity [59]. Similar findings were reported in terms of *H. pylori* and miRNA-146 [60]. Both miRNA-155 and -146a were suggested to be indicators of intestinal metaplasia and follicular gastritis [61]. However, the expression of miRNA-125 was found to be diminished in the setting of *H. pylori* infection [62], which is defined as a potential tumor suppressor [56,63]. Similarly, miRNA-30a also acts as ‘a good guy’ in terms of tumor suppression [60] (Table 1).

Toll-like receptors (TLRs) belong to the family of pathogen recognition receptors, and once their signaling pathways are activated, they will actively contribute to the innate inflammatory responses through the induction of antigen-presenting molecules, chemokines, inflammatory cytokines, and costimulatory molecules [64]. TLR-2 and -4 are the most studied, and their interaction is essential for the premalignant transformation of *H. pylori*-associated chronic inflammation towards metaplasia, dysplasia, and, eventually, gastric adenocarcinoma [65,66]. The aforementioned TLRs are responsible for the recognition of *H. pylori* liposaccharide, which represents the first barrier to bacterial infection [67,68], triggering the synthesis of a wide spectrum of proinflammatory cytokines [69,70]. Their contribution to gastric tumorigenesis is incontestable, as they are strongly related to geographic area, ethnicity and TLRs polymorphisms [20]. In addition, *TLR4* might be used as a targeted receptor to suppress *H. pylori* colonization [71]. *TLR5* has the ability to recognize bacterial flagellin via the p38 *MAP* kinase signaling pathway [20]. Like other *TLRs*, this receptor is also considered a promoter of *H. pylori*-dependent gastric tumorigenesis [20]. *TLR9* recognizes the bacterial DNA of injured host cells and invades microbial agents through its structural components [20,72]. Interestingly, the available evidence on the *TLR-9* function highlighted that, in the setting of *H. pylori* infection, it might have a dichotomous role, acting as either a promotor or a suppressor of this infection depending on the gastric microenvironment [20,72]. Although the research involving other *TLRs*, such as *TLR-1*,*-6*,*-7*,*-8*, and -*10*, is limited, several studies pointed to their implication in the development of *H. pylori*-associated gastropathies, including gastric cancer [20]. In addition to the well-documented partnership between *TLR-2* and *-4*, further studies proved that *TLR-1* and -6 can also form a partnership to support the functioning of TLR-2 [73] (Table 1).

The aberrant methylation of bacterial DNA represents another important consequence of *H. pylori* infection [27]. A recent study pointed out that *H. pylori* is able to methylate almost 2000 positions and more than 400 regions, mostly via being hypermethylated [74]. Contrariwise, Leodolter et al. found that genome-wide hypomethylation was associated with both gastric cancer and *H. pylori*-related high-risk gastritis [75]. Moreover, Liu et al. pointed out, in a study performed on gastric cancer patients, that 55 of them expressed 161 genes that were differentially methylated [76]. The same authors reported that certain genes might be used as prognostic factors in *H. pylori*-positive gastric cancer patients (*CACNB2*, *PREX1*, *MEF2C*, *GNB4*, *GRIN2A*), while others (CACNB2 and MEF2C) predict the overall survival in these patients. Similar findings were reported by other studies regarding the involvement of multiple other genes in DNA methylation induced by *H. pylori,* resulting in a subsequently increased risk of gastric cancer [77,78,79,80,81] (Table 1).

*VacA* and *CagA* represent two major *H. pylori* virulence factors which have been well-established to be related to gastric carcinogenesis mechanism. Both old and new studies proved that individuals harboring CagA-positive *H. pylori* strains experience a higher risk of developing gastric cancer [82,83,84]. The oncoprotein CagA has several complex roles, acting as inductor of inflammation, regulator of autophagy, anti-apoptotic protein, and inhibitor of tumor suppressors though the inhibition of certain pro-apoptotic factors like BIM, SIVA1, and BAD, as well as via the activation of multiple signaling pathways such as WNT/beta-linked protein, PI3K/AKT, RAS/ERK, and JAK/STAT [85,86]. Another signaling pathway that was indicated to be activated by CagA is Ye s-associated protein, resulting in the promotion of the epithelial–mesenchymal transition, which is responsible for the loss of contact between gastric cells, enhancing *H. pylori’s* ability to infiltrate deeper into the infected gastric mucosa [87]. Based on all these findings, CagA is definitely responsible for promoting gastric carcinogenesis. On the other hand, VacA functions as a disruptor of the mucosal barrier within the infected gastric mucosa, interfering with the pathways responsible for antigen presentation, and, at the same time, downregulating phagocytosis, eventually leading to the persistence of *H. pylori* infection [88,89,90]. Moreover, VacA is a partner of CagA due to its ability to downregulate lysosomal degradation and autophagy, which will enable the accumulation of CagA in the epithelial cells of the gastric mucosa [91]. Both the aforementioned virulence factors were deemed the most powerful fighters against eradication therapies. Karbalaei et al. proved in a recently published meta-analysis that CagA-positive strains increase the global resistance to metronidazole, as well as to amoxicillin and levofloxacin, in Western countries [92]. Surprisingly, the authors concluded that VacA-positive strains are associated with reduced antimicrobial resistance. Thus, the VacA s1m1 genotype was proven to decrease the resistance to metronidazole, while VacA s2m2-positive strains were associated with an overall low resistance rate to antibiotics, most likely due to these strains’ ability to induce an anti-inflammatory responses [92] (Table 1).

Lipopolysaccharide is an important component of *H. pylori* and is defined by three major structural domains: O-polysaccharides, core oligosaccharide and lipid A. These are responsible for inhibiting immune responses and enabling the transformation of acute infection to chronic persistent infection [93,94]. The *TLR-4*-dependent pathways are responsible for the relationship between lipopolysaccharide and an increased risk of developing gastric cancer [15,95,96] (Table 1).

**Table 1:** The role of biomarkers in the early detection of premalignant and malignant transformation related to *H. pylori* infection.

Biomarkers	Authors and Year	Effects
Pepsinogens	Kurilovich et al., 2016 [28,29,30,31] & Cai et al., 2016 [28,29,30,31] Lin et al., 2021 [33] Deng et al., 2022 [34]	Predictive power for detecting patients with increased risk of gastric cancer → ratio between pepsinogen I and II. Individuals with gastric cancer → decreased levels of pepsinogen I and pepsinogen I/pepsinogen II ratio. Pepsinogen I < 30 μg/L, pepsinogen II > 30 ng/mL, and pepsinogen I/II ratio < 3 → associated with gastric cancer.
Gastrin 17	Cai et al., 2016 [28,29,30,31] Tu et al., 2017 [30]	Increased level of gastrin → associated with gastric cancer risk. Pepsinogens + *H. pylori* antibodies in the GastroPanel test → the predictive value of gastrin 17 ↑.
Blood glucose	Deng et al., 2022 [34] Lindkvist et al., 2013 [35] Tran et al., 2012 [36] Hidaka et al., 2015 [37]	No association between this parameter and gastric cancer risk [34]. ↑ Blood glucose levels → ↑ risk of gastric carcinoma only in women. ↑ Incidence of gastric cancer in non-smokers, and normal-weight individuals in the absence of family history of gastric neoplasia. Positive correlation between plasma insulin levels, C peptide (only in men), HOMA-IR and gastric cancer.
Lipid metabolism parameters	Asano et al., 2008 [38] Deng et al., 2022 [34]	↓ Serum cholesterol → independent risk factor for gastric carcinoma. No correlation between this parameter and the risk of gastric neoplasia.
Iron-metabolism parameters	Deng et al., 2022 [34]	↓ Ferritin → an early predictor for gastric cancer. serum iron levels, transferrin, and total iron-binding capacity → Associated with gastric cancer risk.
Gastric microecological dysbiosis	Guo et al., 2019 [41,42] & Ferreira et al., 2018 [41,42] Lofgren et al., 2011 [43] Castaño-Rodríguez et al., 2017 [44]	Resident bacteria contribute, along with *H. pylori,* to gastric carcinogenesis → by reducing nitrates → accumulation of N-nitroso and nitrite compounds within the gastric microenvironment, such as *Staphylococcus epidermidis* and *haemolyticus*, *Micrococcus luteus*, *Neisseria mucosa*, *Actinomyces naeslundii* or *Rothia dentocarios.* Commensal bacteria → acceleration of the inflammation produced by *H. pylori*, increasing the risk of gastric carcinogenesis. CagA *H. pylori* interact with *Lactobacillus* → additional inflammatory responses → lactic acid + *H. pylori* → role in gastric carcinogenesis.
*MicroRNAs*	Zhu et al., 2019 [45] Shao et al., 2019 [48] Liu et al., 2012 [50] Qi et al., 2023 [51] Tsai et al., 2020 [53] Huffaker et al., 2017 [59] Cortés-Márquez et al., 2018 [61] Staedel et al., 2013 [62] & Cao et al., 2018 [56,63]	miRNA-425-5p, miRNA-1180-3p, miRNA-7641 and miRNA-122-5p → role in onset of gastric cancer. miRNA-135b-5p → upregulated in tumoral gastric tissues. miRNA-29a/MMP9 → overexpressed in patients with *H. pylori* infection. Upregulation of miRNA-183 → in *H. pylori*-positive gastric cancer patients. Overexpression of miRNA-18a-3p and miRNA-4286 → predict the prognosis of *H. pylori-*induced gastric cancer. Absence of miRNA-155 expression is associated with impaired antitumor immunity. miRNA-155 and -146a → indicators of intestinal metaplasia and follicular gastritis. miRNA-125 → ↓ in the setting of *H. pylori* infection → potential role as a tumor suppressor.
*Toll-like receptors* (TLRs)	Schmausser et al., 2005 [65,66] & Yokota et al., 2010 [65,66] Meliț et al., 2019 [20]	TLR-2 and -4 → role in premalignant transformation of *H. pylori*-associated chronic inflammation towards metaplasia, dysplasia, and eventually gastric adenocarcinoma. *TLR5* → a promoter of *H. pylori*-dependent gastric tumorigenesis.
Aberrant methylation of bacterial DNA	Liu et al., 2020 [76] Sepulveda et al., 2016 [77,78,79,80,81] & Xie et al., 2020 [77,78,79,80,81]	Some genes → role as prognostic factors in *H. pylori*-positive gastric cancer patients (*CACNB2*, *PREX1*, *MEF2C*, *GNB4*, *GRIN2A*), while others (CACNB2 and MEF2C) predict the overall survival in these patients. DNA methylation induced by *H. pylori* → increased risk of gastric cancer.
*VacA* and *CagA*	Nell et al., 2018 [82,83,84] & Palrasu et al., 2020 [85,86]	CagA-positive *H. pylori* strains experience a higher risk of developing gastric cancer → as inductor of inflammation, regulator of autophagy, anti-apoptotic protein, and inhibitor of tumor suppressors though the inhibition of certain pro-apoptotic factors like BIM, SIVA1, and BAD.
Lipopolysaccharide	Wang et al., 2017 [15,95,96]	*TLR-4*-dependent pathways → relationship between lipopolysaccharide and increased risk of developing gastric cancer.

↑: increase, ↓: decrease

### 2.2. H. pylori Infection and Gastric Carcinogenesis Hallmarks in Children

Gastric cancer is a rare condition in pediatric patients, but still possible. Age was found to be a major factor in determining the risk of gastric cancer, with adenocarcinoma being found to have an incidence of 0.1% in pediatric patients [97]. A recent survey performed on Japanese children pointed to an increased prevalence in patients above the age of 10, with 20% of them presenting a positive family background, and the presence of *H. pylori* infection was noted in two out of three children tested for this infection [98]. The role of *H. pylori* infection in the occurrence of intestinal metaplasia, which is defined as a premalignant condition, was also pointed out by Cam et al. in infected Turkish children [99]. A more recent multicenter cross-sectional analysis, including 333 children diagnosed with gastric cancer with a mean age of 11.8 years, identified the presence of *H. pylori* infection in 10.2% of cases [100]. Nevertheless, genetic predisposition is worth mentioning, since it was proved to be present in up to 10% of the cases [100]. A recent study highlighted that the expression of several genes encoding inflammatory molecules that are closely related to the development of gastric cancer, like C-X-C motif chemokine ligand 13, lipocalin-2, regenerating islet-derived 3 alpha, and pim-2, are upregulated even in children with *H. pylori*, not only in adults [101]. Ethnicity was also proved to be related to gastric cancer prevalence, with it being stated that non-Hispanic whites have the most increased risk of developing stomach cancer as compared to African Americans and Asians [100]. Children with gastric solid tumors commonly present with gastrointestinal bleeding, subsequent anemia, dysphagia, abdominal pain, fever, abdominal mass, vomiting, diarrhea, and weight loss [98,100] (Table 2).

The implications of *H. pylori* infection in the development of gastric cancer during adulthood are further sustained by several pediatric studies which pointed out that the eradication of this infection could result in a decrease in stomach malignancies. Thus, studies on children from Japan, where the incidence of gastric cancer is high among adults, revealed that most asymptomatic children who underwent an upper digestive endoscopy were infected with *H. pylori* and presented associated gastropathies such as antral nodular gastritis, atrophy, intestinal metaplasia, or ulcer [102,103]. Moreover, the eradication in these cohorts resulted in an improvement in clinical symptoms like abdominal pain or anemia [102], suggesting that a ‘screen and treat’ strategy in children from Japan could represent a cornerstone of prevention in 6000 people during their adulthood [103]. Similarly, the persistence of this infection in children aged between 3 and 6 years was associated with higher *H. pylori* seroprevalence, a decreased frequency of non-secretor phenotype, and a great variability in gene expression patterns, especially in genes potentially related to carcinogenesis [104]. In older children, within the age range 8–10 years, persistent *H. pylori* infection was proven to significantly increase both the frequency of abdominal pain and the serum levels of pepsinogen II [105]. Similar findings were also reported in Asian and European children [106,107,108,109]. Furthermore, Lucero et al. recently indicated that successful eradication is associated with a decrease in both pepsinogen I and II serum levels [110] (Table 2).

As previously mentioned, gastric microbiota is an important player in the complex process of carcinogenesis. *H. pylori* was proven to trigger important changes in children’s gastric microbial communities by reducing both the abundance and diversity of commensal bacteria such as Firmicutes, Bacteroidetes, Fusobacteria and Actinobacteria [111]. Similar to studies on adults [112,113], the previously mentioned study confirmed that gastric microbiota diversity and abundance can be restored after *H. pylori* eradication [111] (Table 2).

The early diagnosis of gastric damage caused by *H. pylori* infection is definitely the main step in gastric cancer prevention. According to a study from Brazil, children are most commonly infected with highly virulent *H. pylori* strains, increasing the risk of persistent infection and, subsequently, the gastric cancer risk [114]. In fact, the probability of *H. pylori* persistence in children was reported to vary between 49 and 95% [115]. Due to this long-term persistence, a cascade of events occurs favoring the transformation of premalignant lesions into gastric cancer, with gastric atrophy being the prototype of the first step in this process, since it was stated that this will result in malignant carcinoma, while non-atrophic gastritis was associated with benign lesions [116]. Moreover, recent findings strengthened our hypothesis that gastric cancer roots occur during childhood, proving that precancerous lesions such as gastric atrophy, intestinal metaplasia and spasmolytic peptide-expressing metaplasia affect approximately 30.4% of infected children, with prevalence rates varying between 4.3% for intestinal metaplasia and 30.4% for gastric glandular atrophy [117]. A larger study from China pointed to an overall infection rate in pediatric patients of 84.14%, with an incidence of precancerous lesions of 4.33% accounting for atrophic gastritis, intestinal metaplasia and dysplasia [118] (Table 2).

The importance of detecting gastric damage biomarkers in children was recently emphasized by George et al., who noticed that several genes and/or proteins identified in adults with gastric cancer were also differentially expressed/hypermethylated in *H. pylori*-infected children [119]. Nanomechanical properties of the gastric tissue induced by *H. pylori* infection were also stated to contribute to the future development of gastric cancer [120]. Thus, Deptula et al. performed a study on *H. pylori-*infected children and noticed that infected gastric tissues are softer than healthy ones, and the continuous changes in tissue mechanical properties triggered by this infection might be a promoter of gastric cancer [120] (Table 2).

Aberrant immune system responses were also found to be present in children with *H. pylori* infection. Although limited studies have been performed ton this topic to date, several authors emphasized the role of *H. pylori* in modifying the host’s immune responses to this infection. Thus, Helmin-Basa recently underlined the lack of mature CD83+ in the gastric tissue samples of children with *H. pylori* infection, hypothesizing that this peculiarity might enable tolerance to local antigens, decreasing the inflammatory responses [121]. Previous studies of our team supported the implications of innate immunity in promoting or suppressing *H. pylori* infection in children, revealing the contribution of certain *TLR* polymorphisms like *TLR2 rs3804099* and *TLR9 rs352140*, as well as of inflammasomes (*NLRP3 rs10754558*), in augmenting inflammation as a response to this infection [2] (Table 2).

Despite the scarcity of evidence regarding the relationship between *H. pylori* infection and gastric carcinogenesis in children, the alarming aforementioned evidence suggests that this is a reality, and that gastric carcinogenesis might have a very early onset during childhood.

**Table 2:** Gastric carcinogenesis hallmarks in children.

Risk Factors	Authors and Year	Effects
Age	Tessler est al, 2019 [97] Okuda et al., 2019 [98]	A major factor → adenocarcinoma → an incidence of 0.1% in pediatric patients. Children aged >10 years → 20% of them have a positive family background and the presence of *H. pylori* infection was higher.
*H. pylori* infection	Cam et al. [99] Attard et al., 2023 [100] Braga et al., 2014 [114] Hsieh et al., 2022 [117] Yu et al., 2022 [118] Honma et al., 2019 [102]	Intestinal metaplasia. Children with gastric cancer mean aged 11.8 years → *H. pylori* infection was present in 10.2% of the cases. Children are most commonly infected with highly virulent *H. pylori* strains, increasing the risk of persistent infection and gastric cancer. Precancerous lesions such as gastric atrophy, intestinal metaplasia and spasmolytic peptide-expressing metaplasia affect 30.4% of infected children → prevalence rates between 4.3% for intestinal metaplasia and 30.4% for gastric glandular atrophy. In China → overall infection rate in pediatric patients—84.14%, with an incidence of precancerous lesions of 4.33%. In Japan → higher incidence of gastric cancer in children infected with *H. pylori*.
Genetic predisposition	Attard et al., 2023 [100] Obayashi et al., 2016 [101]	Up to 10% of cases. Several genes encoding inflammatory molecules → development of gastric cancer like C-X-C motif chemokine ligand 13, lipocalin-2, regenerating islet-derived 3 alpha, and pim-2 are upregulated even in children with *H. pylori*.
Ethnicity	Attard et al., 2023 [100]	Non-Hispanic whites → increased risk of developing stomach cancer.
Gastric microbiota	Miao et al., 2020 [111]	An important player in the complex process of carcinogenesis → *H. pylori* is a modulator of children’s gastric microbial communities by reducing the abundance and diversity of commensal bacteria (Firmicutes, Bacteroidetes, Fusobacteria and Actinobacteria).
Damage biomarkers	George et al., 2020 [119] Deptuła et al., 2021 [120]	Several genes and/or proteins → differentially expressed/hypermethylated in *H. pylori*-infected children. Nanomechanical properties of the gastric tissue induced by *H. pylori* infection → contribute to the development of gastric cancer.
Aberrant immune system responses	Helmin-Basa et al., 2019 [121] Meliț LE et al., 2022 [2]	The lack of mature CD83+ in the gastric tissue samples of children with *H. pylori* infection → this peculiarity might enable tolerance to local antigens, decreasing the inflammatory responses. Innate immunity → promotes or suppresses *H. pylori* infection in children, revealing the contribution of certain *TLR* polymorphisms like *TLR2 rs3804099* and *TLR9 rs352140*, as well as of inflammasomes (*NLRP3 rs10754558*), in augmenting inflammation.

## 3. Conclusions

Gastric cancer is extremely rare in children and, if present, usually has a hereditary pattern. Nevertheless, *H. pylori* infection, which is well-documented to be one of the main factors involved in gastric carcinogenesis, is commonly acquired during childhood and can persist for life. Its long-term persistence is enabled by the interaction between *H. pylori* virulence and host-related factors, with the latter being the result of the local and systemic changes induced by the bacterium itself. *H. pylori* is thus able to trick the host into being its partner by promoting the persistence of this infection instead of fighting against it. The timely diagnosis and eradication of this infection are the only things that can preempt the premalignant and further malignant transformation of normal gastric epithelium. Taking into account that this is a long-standing process, lasting from childhood into adulthood, further research should focus on identifying the precise onset of tumorigenesis process in the setting of *H. pylori* during childhood.

## Data Availability

Not applicable.
